# Effects of greenhouse gases and hypoxia on the population of aquatic species: a fractional mathematical model

**DOI:** 10.1186/s13662-022-03679-8

**Published:** 2022-04-15

**Authors:** Pushpendra Kumar, V. Govindaraj, Vedat Suat Erturk, Mohamed S. Mohamed

**Affiliations:** 1 Department of Mathematics, National Institute of Technology Puducherry, Karaikal, 609609 India; 2 Department of Mathematics, Faculty of Arts and Sciences, Ondokuz Mayis University, Atakum, 55200 Samsun Turkey; 3 Department of Mathematics and Statistics, College of Science, Taif University, P.O. Box 11099, Taif, 21944 Saudi Arabia

**Keywords:** 26A33, 65D05, 65D30, 65L07, 92B05, Dissolved oxygen, Temperature, Aquatic species, Greenhouse gases, Fractional mathematical model, Numerical method, Modified Caputo fractional derivative

## Abstract

Study of ecosystems has always been an interesting topic in the view of real-world dynamics. In this paper, we propose a fractional-order nonlinear mathematical model to describe the prelude of deteriorating quality of water cause of greenhouse gases on the population of aquatic animals. In the proposed system, we recall that greenhouse gases raise the temperature of water, and because of this reason, the dissolved oxygen level goes down, and also the rate of circulation of disintegrated oxygen by the aquatic animals rises, which causes a decrement in the density of aquatic species. We use a generalized form of the Caputo fractional derivative to describe the dynamics of the proposed problem. We also investigate equilibrium points of the given fractional-order model and discuss the asymptotic stability of the equilibria of the proposed autonomous model. We recall some important results to prove the existence of a unique solution of the model. For finding the numerical solution of the established fractional-order system, we apply a generalized predictor–corrector technique in the sense of proposed derivative and also justify the stability of the method. To express the novelty of the simulated results, we perform a number of graphs at various fractional-order cases. The given study is fully novel and useful for understanding the proposed real-world phenomena.

## Introduction

In the study of greenhouse effects, we know that in the day the sun warms up the atmosphere of earth. But when the Earth supercools at the night, then the presented heat is radiated again into the environment. In the duration of this process, the heat is exploited by the greenhouse gases in the environment of earth. This process makes the layer of the earth thermal, which causes the possibility of living being’s survival on earth. However, because of the increment in the level of greenhouse gases, the earth’s temperature has raised simultaneously. This has caused a number of drastic impacts. In the list of reasons of greenhouse effect, deforestation, burning of fossil fuels, farming, industrial waste, and landfills play a major role. The major effects of increased greenhouse gases are depletion of ozone layer, global warming, air and smog pollution, water bodies acidification, etc. Since the starting of the industrial revolution, the concentration of carbon dioxide, chlorofluorocarbon (CFC), nitrous oxide, and methane have enhanced in the environment, and there is firm witness that the venomous impacts of greenhouse gases on our ecological systems have been taken account as a outcome of human bustles.

**Figure 1:**
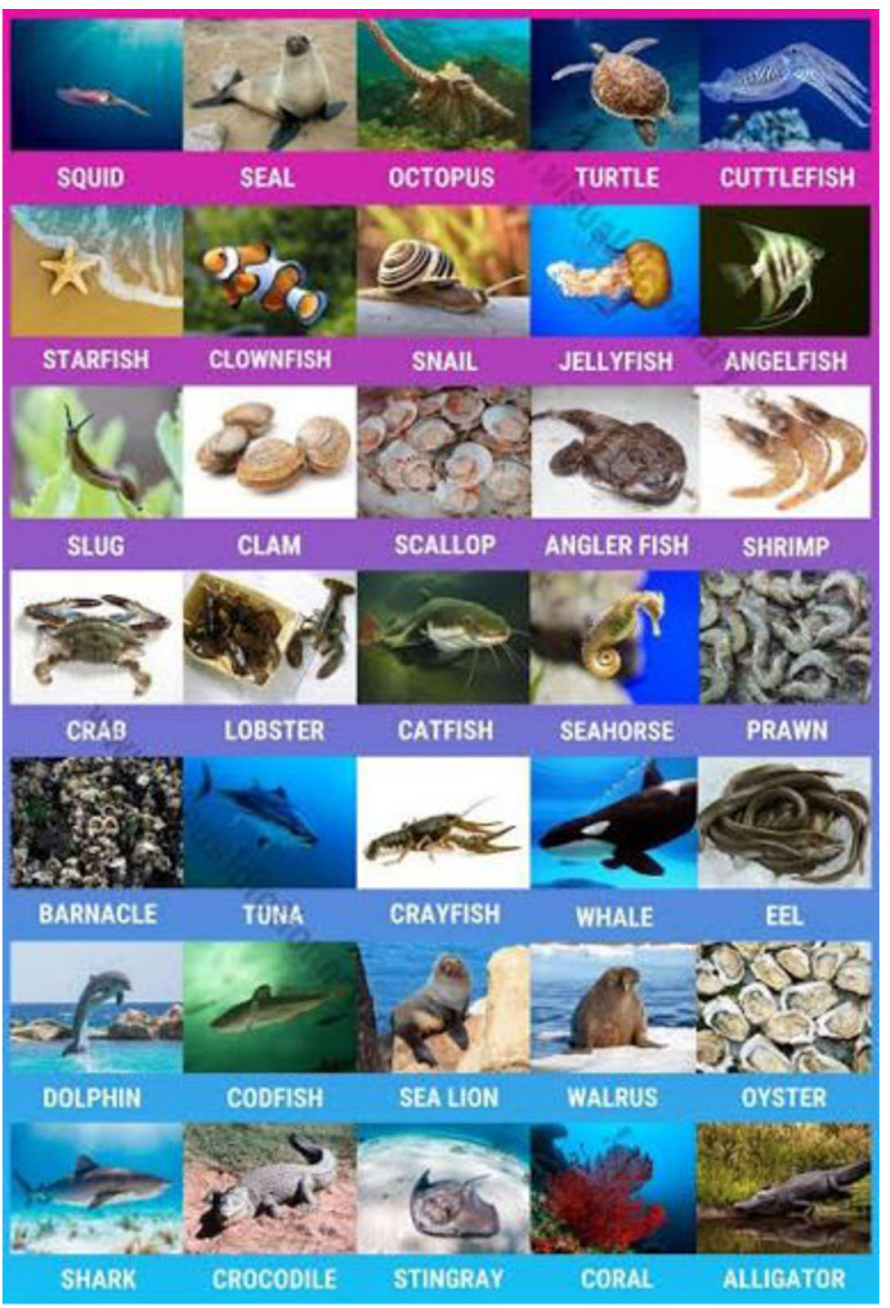
Some aquatic animals

Aquatic life simply means to stay in surface water, and water in this paragon is specified as a marine habitat. Living beings that live in the water either permanently or momentarily are called aquatic animals and plants, and these compose the beings in water aquatic life. It is well known that the increment in the temperature of water causes the reduction in the concentration of mixed oxygen of the aquatic environment and also rises the requirement of mixed oxygen for the aquatic animals. Invertebrates, fish, and other aquatic species rely upon the amount of oxygen decomposed in the water, and in the absence of it, they may not live. A small changes in concentration of mixed oxygen can effect the conformation of aquatic society [1]. So the rate of survival of the aquatic density (Fig. 1) goes down under hypoxia, and the oxygen necessary for their living raises with growth in temperature [2, 3]. Hence, because of the combined influences of reduced concentration of mixed oxygen and enhanced demand of oxygen by the animals, the warming of species bodies rises the death rate of species population [3, 4]. To define these dynamics, a number of models have been proposed, but only few models [5, 6] have been given to simulate the effects of dissolved oxygen and temperature on the population of aquatic species.

In the matter of the above discussion, in this study, we prepare a fractional-order mathematical system to simulate the joint influences of low mixed oxygen density, exalted water temperature, and raised oxygen demand on the extinction or survival of aquatic population. Fractional derivatives are one of the most effective tools for simulations and have been proposed in many different ways (for instance, see [7–9]). Fractional-order models have been widely used to define a number of real-world problems because their memory effects make these models more visible in the literature. Recently, a number of fractional-order models have been prepared by researchers. In this regard, in [10–18] the authors have proposed a number of fractional-order mathematical models to describe the dynamics of Covid-19 epidemic. In [19, 20] the authors have simulated the fractional-order dynamics of well-known lassa hemorrhagic fever. The applications of fractional derivatives in ecology can be seen in [21]. Regarding some more specific areas, nonclassical derivatives have been successfully used to derive the structure of tuberculosis [22], malaria [23], mosaic disease [24], Nipah epidemic [25], canine distemper virus [26], and huanglongbing transmission [27]. In [28] the authors used a fractional-order time-delay mathematical model to describe the process of oncolytic virotherapy. A study on analytic solution for oxygen diffusion from capillary to tissues via fractional derivatives is proposed in [29]. Also, an application of a new generalized Caputo derivative to define the famous love story of Layla and Majnun is given in [30]. So the literature of fractional-order calculus is increasing exponentially day by day. Also, a number of true and false results come on the various fractional derivatives. Recently, in [31] the authors have proved that in the case of evolution equations in terms of the Caputo–Fabrizio and Atangana–Baleanu fractional derivatives, intrinsic discontinuities occur. The geometry of fractional-order derivatives is still not well-defined, but their applications in different scientific fields make them more visible to the literature. Some important studies related to the properties of fractional derivatives, special functions, and different types of inequalities can be learned from [32–35]. Nonstandard Chebyshev collocation and finite difference schemes for solving fractional diffusion equations are proposed in [36]. Some novel analysis on the fractional differential equations for the generalized Mittag-Leffler function are discussed in [37]. A study on the analytical solutions of the fractional-order equations with uncertainty is proposed in [38]. Alderremy et al. [39] have discussed some novel models of the multispace-fractional Gardner equation. A study on spectral collocation method for solving smoking model is proposed in [25]. In [40] the authors have proposed a study on Darcy–Brinkman–Forchheimer model for nanobioconvection stratified MHD flow through an elastic surface. In [41] a reduced differential transform scheme for simulating nonlinear biomathematics models is given. In [42] a study on dynamical features and signal flow graph of nonlinear noninteger order smoking mathematical model is explored. In [43], some numerical methods for a model of relativistic electrons arising in the laser thermonuclear fusion are investigated. The manuscript is designed as follows: In Sect. 2, firstly, we remind some important definitions and results. In Sect. 3, we give a complete description of the proposed fractional-order nonlinear model, where we define the significance and importance of every small part of the model. Then in Sect. 4, we give a complete mathematical analysis related to the solution existence, derivation, and stability. To show the correctness of our results, in Sect. 5, we present the necessary graphs at various fractional-order values and parameter weights. At the end, a conclusion gives a comfortable end to the paper.

## Preliminaries

Firstly, we remind some important definitions and results.

### Definition 1

([44])

The new definition of the Caputo-type fractional derivative $D^{\sigma, \varkappa }_{d_{+}}$ of order $\sigma >0$ (called a new generalized Caputo) for the function $\Psi \in C^{1}([d, T])$ is given by

1 $$\begin{aligned} \bigl(D^{\sigma, \varkappa }_{d_{+}} \Psi \bigr) (\xi ) = \frac{\varkappa ^{\sigma - n+ 1}}{\Gamma (n- \sigma ) } \int ^{\xi }_{d}{s^{ \varkappa - 1}{\bigl(\xi ^{\varkappa }- s^{\varkappa }\bigr)}^{n- \sigma - 1} \biggl({s^{1- \varkappa } \frac{d}{ds}} \biggr)^{n}\Psi (s)\,ds}, \quad\xi > d, \end{aligned}$$

where $\rho > 0$, $d \geq 0$, *and* $n- 1< \sigma \leq n$.

### Lemma 1

([45])

*For* $0 < b < 1$ *and a nonnegative integer* *ϱ*, *there exist positive constants* $\mathcal{C}_{b, 1}$ *and* $\mathcal{C}_{b, 2}$, *dependent only on* *b*, *such that*

$$\begin{aligned} (\varrho + 1)^{b}- \varrho ^{b} \leq \mathcal{C}_{b, 1}( \varrho + 1)^{b- 1} \end{aligned}$$

*and*

$$\begin{aligned} (\varrho + 2)^{b+ 1} - 2(\varrho + 1)^{b+ 1} + \varrho ^{b+ 1} \leq \mathcal{C}_{b, 2}(\varrho + 1)^{b - 1}. \end{aligned}$$

### Lemma 2

([45])

*Let* $d_{q, s} = (s - q )^{b- 1}$ *for* $q = 1, 2,\ldots, s - 1$ *and* $d_{q, s}= 0$ *for* $q \geq s$, *let* $M, b, h, T> 0$, *and* $rh\leq T$, *where* *r* *is a positive integer*. *Let* $\sum_{q= r}^{q= s}d_{q, s}|e_{q}|= 0$ *for* $r> s \geq 1$. *If*

$$\begin{aligned} \vert e_{s} \vert \leq Mh^{b} \sum _{q=1}^{s-1}d_{q,s} \vert e_{q} \vert + \vert \beta _{0} \vert ,\quad s= 1,2,\ldots,r, \end{aligned}$$

*then*

$$\begin{aligned} \vert e_{r} \vert \leq \mathcal{C} \vert \beta _{0} \vert ,\quad r= 1,2,\ldots, \end{aligned}$$

*where* $\mathcal{C}$ *is a positive constant not dependent on* *r* *and* *h*.

## Model dynamics

Now we propose a fractional-order mathematical model to study the proposed dynamics. In [1] the authors have already given an idea on the proposed topic by using an integer-order model. We propose a fractional-order model because it is well known that the memory effects, which cannot be studied in the classical case, can be easily observed by fractional-order derivatives. It is very important that when we propose a fractional-order model, it should have the same time dimension on both sides of the system. Taking care of all these aspects, we define the novel fractional-order model as follows:

2 $$\begin{aligned} \textstyle\begin{cases} {}^{C}D_{t}^{\sigma, \varkappa } N(t) = G(U, T)N- \frac{g_{0}^{\sigma }N^{2}}{\gamma _{0}}, \\ {}^{C}D_{t}^{\sigma, \varkappa } T(t) = w^{\sigma }C- \zeta _{1}(T- T_{10})+ \gamma ^{\sigma }(Z_{0}- Z), \\ {}^{C}D_{t}^{\sigma, \varkappa } C(t) = A_{0}- \delta _{1}^{\sigma }C, \\ {}^{C}D_{t}^{\sigma, \varkappa } Z(t) = O_{c}^{\sigma }- \Lambda _{1}^{\sigma }Z- \Lambda ^{\sigma }Z C, \\ {}^{C}D_{t}^{\sigma, \varkappa } U(t) = \gamma _{1} \beta ^{(T- T_{0})}(D_{s}(T)- U)- \delta _{2}^{\sigma }UN- \zeta ^{\sigma }(T- T_{\mathrm{opt}}), \end{cases}\displaystyle \end{aligned}$$

where ${}^{C}D_{t}^{\sigma, \varkappa }$ is the new generalized Caputo-type fractional-order operator of order *σ*. In this model,

$$\begin{aligned} &G(U, T)= g_{0}^{\sigma } \biggl(\exp \biggl( -b \biggl( \frac{T- T_{\mathrm{opt}}}{T_{\max }- T_{\mathrm{opt}}} \biggr) \biggr)+ \biggl( \frac{U- U_{0}(T)}{U+1} \biggr) \biggr),\\ &U_{0}(T)= \beta ^{\sigma }_{10}+ \beta ^{\sigma }_{11}(T- T_{\mathrm{opt}}), \end{aligned}$$

and

$$\begin{aligned} D_{s}(T)= \frac{D_{s_{0}}}{1+ T- T_{\mathrm{opt}}}. \end{aligned}$$

In this model, we have five different classes, in which class *N* shows the logistically crescent aquatic species density whose rate of growth is taken as a function of temperature and mixed oxygen, *U* justifies the dissolved oxygen concentrations, *T* defines the water temperature average of the species, *C* expresses the greenhouse gases accumulative concentrations, and *Z* justifies the concentration of ozone. Also, the term $G(U, T)$ expresses the specific rate of growth of the species, which is in fact an exponentially decreasing function of *T* for $T > T_{\mathrm{opt}}$ and increasing function of *U*. The function $U_{0}(T)$ denotes the quantity of dissolved oxygen demanded by species population, which rises with temperature increase.

The term $D_{s}(T)$ is defined for the consideration that if the water temperature level is high, close to the optimum temperature, then the natural loaded dissolved oxygen concentration reduces. The significance of all other parameters is completely given in Table 1. The more deep texture of the given model in classical sense can be learned from [1].

**Table 1:** Description of model parameters

$g_{0}$	Intrinsic growth rate
$\beta _{10}$	Dissolved oxygen’s minimum natural concentration needed by the aquatic species
$\beta _{11}$	Increment rate in the mass of mixed oxygen demanded for the species per unit rise in the level of temperature above the suitable temperature
$T_{\mathrm{opt}}$	Optimal water temperature for the aquatic species maximum rate of growth
$\gamma _{0}$	Carrying capacity of the environment
$D_{s_{0}}$	Dissolved oxygen’s natural saturated concentration at $T = T_{\mathrm{opt}}$
$A_{0}$	Ejection rate of greenhouse gases cause of anthropogenic bustles
*w*	Increment rate in the temperature of water cause of greenhouse gases
$\zeta _{1}$	Coefficient of heat transfer of surface
$O_{c}$	Physic manufacture of concentration of ozone per unit time in the environment
$\Lambda _{1}$	Natural deterioration rate of concentration of ozone
Λ	Deterioration rate of concentration of ozone cause of greenhouse gases
$\gamma _{1}$	Coefficient of reaeration at the reference temperature
$\delta _{2}$	Deterioration rate of mixed oxygen because of breathing by the species
*ζ*	Deterioration rate of mixed oxygen because of a rise in the temperature above the suitable temperature
*β*	A constant that succumbs upon the tincture state of the water body
*γ*	Variations rate in the water temperature because of changes in the ozone concentration level associated with its threshold value
$Z_{0}$	Threshold of concentration of ozone below which temperature will rise
$T_{10}$	Temperature of the environment
$\delta _{1}$	Depletion rate of greenhouse gases
$T_{0}$	Context temperature (associated with the turbulence degree in the water, in which turn succumbs on the depth and speed of the river)
*b*	Constant that incarnates the toxic influence of divergence of *T* from $T_{\mathrm{opt}}$ and divergence of *T* from $T_{\max }$
$T_{\max }$	Maximum temperature of water at which growth can occur
*N*(0)	Initial population of *N*
*T*(0)	Initial population of *T*
*C*(0)	Initial population of *C*
*Z*(0)	Initial population of *Z*
*U*(0)	Initial population of *U*

The equilibria of the given fractional-order mathematical model can be obtained by solving the following system:

3 $$\begin{aligned} &G(U, T)N- \frac{g_{0}^{\sigma }N^{2}}{\gamma _{0}}= 0, \end{aligned}$$

4 $$\begin{aligned} &w^{\sigma }C- \zeta _{1}(T- T_{10})+ \gamma ^{\sigma }(Z_{0}- Z)= 0, \end{aligned}$$

5 $$\begin{aligned} &A_{0}- \delta _{1}^{\sigma }C= 0, \end{aligned}$$

6 $$\begin{aligned} &O_{c}^{\sigma }- \Lambda _{1}^{\sigma }Z- \Lambda ^{\sigma }Z C= 0, \end{aligned}$$

7 $$\begin{aligned} &\gamma _{1} \beta ^{(T- T_{0})}\bigl(D_{s}(T)- U\bigr)- \delta _{2}^{\sigma }UN- \zeta ^{\sigma }(T- T_{\mathrm{opt}})= 0. \end{aligned}$$

Equation (5) gives

8 $$\begin{aligned} C= \frac{A_{0}}{\delta _{1}^{\sigma }}. \end{aligned}$$

Equation (6) gives

9 $$\begin{aligned} Z= \frac{O_{c}^{\sigma }}{\Lambda _{1}^{\sigma }+ \Lambda ^{\sigma }C}. \end{aligned}$$

Equation (4) gives

10 $$\begin{aligned} T= \frac{w^{\sigma }C+ \zeta _{1} T_{10}+ \gamma ^{\sigma }(Z_{0}- Z)}{\zeta _{1}}. \end{aligned}$$

Here we have two different types of equilibrium points.

1. Boundary equilibrium point $\bar{E}= (\bar{U}, \bar{Z}, \bar{C}, \bar{T}, \bar{N})$:

$\bar{N}=0$ (no species population), $\bar{U}= ( \frac{D_{s_{0}}}{1+\bar{T}-T_{\mathrm{opt}}}- \frac{\zeta ^{\sigma }(\bar{T}-T_{\mathrm{opt}})}{\gamma _{1} \beta ^{(\bar{T}-T_{0})}} )$. Here $\bar{C}, \bar{Z}, \bar{T}$ are given by (8), (9), (10), respectively.

A boundary equilibrium point *Ē* exists if $D_{s_{0}} \gamma _{1} \beta ^{(\bar{T}-T_{0})} - \zeta ^{\sigma }( \bar{T}-T_{\mathrm{opt}})(1+\bar{T}-T_{\mathrm{opt}})> 0$ and $Z_{0} > \bar{Z}$.

2. Interior equilibrium point $E^{*}(U^{*}, Z^{*}, C^{*}, T^{*}, N^{*})$, where $N^{*}= \gamma _{0} (\exp (-b ( \frac{T^{*}-T_{\mathrm{opt}}}{T_{\max } - T_{\mathrm{opt}}} ) )+ \frac{U^{*}-(\beta _{10}^{\sigma }+\beta _{11}^{\sigma }(T^{*}-T_{\mathrm{opt}}))}{1+U^{*}} )$ (species population exists) and $N^{*}> 0$, provided that $U^{*}-(\beta _{10}^{\sigma }+\beta _{11}^{\sigma }(T^{*}-T_{\mathrm{opt}}))> 0$. Here $C^{*}, Z^{*}, T^{*}$ are given by Equations (8), (9), (10), respectively, and $U^{*}$ is the positive root of the quadratic equation

11 $$\begin{aligned} a_{1} U^{*^{2}}+ b_{1}U^{*} + c_{1}= 0, \end{aligned}$$

where $a_{1}= \gamma _{1} \beta ^{\sigma (T^{*}- T_{0})}(1+ T^{*}- T_{ \mathrm{opt}})+ \delta _{2}^{\sigma }\gamma _{0} \exp (-b ( \frac{T^{*}-T_{\mathrm{opt}}}{T_{\max } - T_{\mathrm{opt}}} ) )(1+T^{*}-T_{\mathrm{opt}})+ \delta _{2}^{\sigma }\gamma _{0} (1+ T^{*}- T_{\mathrm{opt}})$, $b_{1}= \gamma _{1} \beta ^{(T^{*}- T_{0})}(1+ T^{*}- T_{\mathrm{opt}})- \gamma _{1}\beta ^{(T^{*}- T_{0})}D_{s_{0}}+ \delta _{2}^{\sigma }\gamma _{0} \exp (-b ( \frac{T^{*}-T_{\mathrm{opt}}}{T_{\max } - T_{\mathrm{opt}}} ) )(1+T^{*}-T_{\mathrm{opt}})+ \delta _{2} \gamma _{0} (1+ T^{*}- T_{ \mathrm{opt}})(\beta _{10}^{\sigma }+\beta _{11}^{\sigma }(T^{*}-T_{ \mathrm{opt}}))+ \zeta ^{\sigma }(T^{*}-T_{\mathrm{opt}})(1+ T^{*}- T_{ \mathrm{opt}})$, $c_{1}= \zeta ^{\sigma }(T^{*}-T_{\mathrm{opt}})(1+ T^{*}- T_{ \mathrm{opt}})- \gamma _{1}\beta ^{(T^{*}- T_{0})}D_{s_{0}}$. When the given conditions are taken account, the quadratic equation (11) has at least one positive root if $a_{1}>0, b_{1}>0$, and $c_{1}<0$. Now we derive the following nonautonomous system after solving the given model (2) for *C*:

12 $$\begin{aligned} &{}^{C}D_{t}^{\sigma, \varkappa } N(t) = G(U, T)N- \frac{g_{0}^{\sigma }N^{2}}{\gamma _{0}}, \end{aligned}$$

13 $$\begin{aligned} &{}^{C}D_{t}^{\sigma, \varkappa } U(t) = \gamma _{1} \beta ^{(T- T_{0})}\bigl(D_{s}(T)- U\bigr)- \delta _{2}^{\sigma }UN- \zeta ^{\sigma }(T- T_{\mathrm{opt}}) , \end{aligned}$$

since $Z^{*} \leq \lim_{t \rightarrow \infty } \sup Z(t), C^{*} \leq \lim_{t \rightarrow \infty } \sup C(t), T^{*} \leq \lim_{t \rightarrow \infty } \sup T(t)$.

Hence the fractional-order nonautonomous model (12)–(13) can be specified in the following equivalent fractional-order autonomous model:

14 $$\begin{aligned} &{}^{C}D_{t}^{\sigma, \varkappa } N(t) = g_{0}^{\sigma } \biggl(\exp \biggl(-b \biggl( \frac{T^{*}-T_{\mathrm{opt}}}{T_{\max } - T_{\mathrm{opt}}} \biggr) \biggr)+ \frac{U-(\beta _{10}^{\sigma }+\beta _{11}^{\sigma }(T^{*}-T_{\mathrm{opt}}))}{1+U} \biggr) \\ &\phantom{{}^{C}D_{t}^{\sigma, \varkappa } N(t) = }{} -N- \frac{g_{0}^{\sigma }N^{2}}{\gamma _{0}}, \end{aligned}$$

15 $$\begin{aligned} &{}^{C}D_{t}^{\sigma, \varkappa } U(t) = \gamma _{1} \beta ^{(T^{*}- T_{0})} \biggl( \frac{D_{s_{0}}}{1+T^{*}-T_{\mathrm{opt}}}-U \biggr)- \delta _{2}^{\sigma }UN- \zeta ^{\sigma }\bigl(T^{*}- T_{\mathrm{opt}}\bigr). \end{aligned}$$

The equilibrium points of the dynamic system (14)–(15) are calculated by the following group of equations:

16 $$\begin{aligned} &g_{0}^{\sigma } \biggl(\exp \biggl(-b \biggl( \frac{T^{*}-T_{\mathrm{opt}}}{T_{\max } - T_{\mathrm{opt}}} \biggr) \biggr)+ \frac{U-(\beta _{10}^{\sigma }+\beta _{11}^{\sigma }(T^{*}-T_{\mathrm{opt}}))}{1+U} \biggr) -N- \frac{g_{0}^{\sigma }N^{2}}{\gamma _{0}}= 0, \end{aligned}$$

17 $$\begin{aligned} &\gamma _{1} \beta ^{(T^{*}- T_{0})} \biggl( \frac{D_{s_{0}}}{1+T^{*}-T_{\mathrm{opt}}}-U \biggr)- \delta _{2}^{\sigma }UN- \zeta ^{\sigma } \bigl(T^{*}- T_{\mathrm{opt}}\bigr)= 0. \end{aligned}$$

1. Boundary equilibrium point $\bar{\bar{E}}(\bar{\bar{U}}, \bar{\bar{N}})$:

$$\begin{aligned} &\bar{\bar{N}}= 0 \quad(\text{no species population}),\\ &\bar{\bar{U}}= \biggl(\frac{D_{s_{0}}}{1+T^{*}-T_{\mathrm{opt}}}- \frac{\zeta ^{\sigma }(T^{*}- T_{\mathrm{opt}})}{\gamma _{1} \beta ^{(T^{*}- T_{\mathrm{opt}})}} \biggr). \end{aligned}$$

The existence of boundary equilibrium point $\bar{\bar{E}}$ provides

$$\begin{aligned} D_{s_{0}} \gamma _{1} \beta ^{(T^{*}- T_{\mathrm{opt}})}- \zeta ^{\sigma }\bigl(1+T^{*}-T_{ \mathrm{opt}}\bigr) \bigl(T^{*}- T_{\mathrm{opt}}\bigr)> 0. \end{aligned}$$

2. Interior equilibrium point $E^{**}(U^{**}, N^{**})$:

$N^{**}= \gamma _{0} (\exp (-b ( \frac{T^{*}-T_{\mathrm{opt}}}{T_{\max } - T_{\mathrm{opt}}} ) )+ \frac{U^{**}-(\beta _{10}^{\sigma }+\beta _{11}^{\sigma }(T^{*}-T_{\mathrm{opt}}))}{1+U^{**}} )$ (aquatic population exists) and $N^{**}> 0$, provided that

$$\begin{aligned} U^{**}-\bigl(\beta _{10}^{\sigma }+\beta _{11}^{\sigma }\bigl(T^{*}-T_{\mathrm{opt}}\bigr) \bigr)> 0, \end{aligned}$$

where $U^{**}$ is a positive root of the quadratic equation

18 $$\begin{aligned} A_{1} U^{*^{2}}+ B_{1}U^{*} + C_{1}= 0, \end{aligned}$$

where $A_{1}= \gamma _{1} \beta ^{(T^{*}- T_{0})}(1+T^{*}-T_{\mathrm{opt}})+ \delta _{2}^{\sigma }\gamma _{0} \exp (-b ( \frac{T^{*}-T_{\mathrm{opt}}}{T_{\max } - T_{\mathrm{opt}}} ) )(1+T^{*}-T_{\mathrm{opt}})+ \delta _{2}^{\sigma }\gamma _{0} (1+T^{*}-T_{ \mathrm{opt}})$, $B_{1}= \gamma _{1} \beta ^{(T^{*}- T_{0})}(1+T^{*}-T_{\mathrm{opt}})- \gamma _{1} \beta ^{(T^{*}- T_{0})}D_{s_{0}}+ \delta _{2}^{\sigma }\gamma _{0} \exp (-b ( \frac{T^{*}-T_{\mathrm{opt}}}{T_{\max } - T_{\mathrm{opt}}} ) )(1+T^{*}-T_{\mathrm{opt}})- \delta _{2}^{\sigma }\gamma _{0} (1+T^{*}-T_{\mathrm{opt}})( \beta _{10}^{\sigma }+\beta _{11}^{\sigma }(T^{*}-T_{\mathrm{opt}}))+\zeta ^{\sigma }(T^{*}-T_{\mathrm{opt}})(1+T^{*}-T_{\mathrm{opt}})$, $C_{1}= \zeta ^{\sigma }(T^{*}-T_{\mathrm{opt}})(1+T^{*}-T_{ \mathrm{opt}})- \gamma _{1} \beta ^{(T^{*}- T_{0})}D_{s_{0}}$.

If the given constraints are satisfied, then the quadratic equation specified by (18) has at least one positive root if

$$\begin{aligned} A_{1}> 0,\qquad B_{1}> 0,\qquad C_{1}< 0. \end{aligned}$$

### Lemma 3

*For the fractional*-*order mathematical system* (14)*–*(15), $\bar{\bar{E}}$ *is locally asymptotically stable if* $g_{0}^{\sigma } (\exp (-b ( \frac{T^{*}-T_{\mathrm{opt}}}{T_{\max } - T_{\mathrm{opt}}} ) )+ \frac{\bar{\bar{U}}-(\beta _{10}^{\sigma }+\beta _{11}^{\sigma }(T^{*}-T_{\mathrm{opt}}))}{1+\bar{\bar{U}}} )< 0$ *and is an unstable saddle point if* $g_{0}^{\sigma } (\exp (-b ( \frac{T^{*}-T_{\mathrm{opt}}}{T_{\max } - T_{\mathrm{opt}}} ) )+ \frac{\bar{\bar{U}}-(\beta _{10}^{\sigma }+\beta _{11}^{\sigma }(T^{*}-T_{\mathrm{opt}}))}{1+\bar{\bar{U}}} )> 0$.

### Proof

After the linearization, taking the Laplace transform of both sides of system (14)–(15), the Jacobian matrix for system (14)–(15) simulated at $\bar{\bar{E}}$ is given by

$$\begin{aligned} M_{11}= \begin{bmatrix} a_{11} & 0 \\ a_{21} & a_{22}\end{bmatrix}, \end{aligned}$$

where

$$\begin{aligned} a_{11}= g_{0}^{\sigma } \biggl(\exp \biggl(-b \biggl( \frac{T^{*}-T_{\mathrm{opt}}}{T_{\max } - T_{\mathrm{opt}}} \biggr) \biggr)+ \frac{\bar{\bar{U}}-(\beta _{10}^{\sigma }+\beta _{11}^{\sigma }(T^{*}-T_{\mathrm{opt}}))}{1+\bar{\bar{U}}} \biggr), \end{aligned}$$

$a_{21}= -\delta _{2} \bar{\bar{U}}, a_{22}= -\gamma _{1} \beta ^{(T^{*}- T_{0})}$. The eigenvalues associated with the matrix $M_{11}$ are $\lambda _{1}=g_{0}^{\sigma } (\exp (-b ( \frac{T^{*}-T_{\mathrm{opt}}}{T_{\max } - T_{\mathrm{opt}}} ) )+ \frac{\bar{\bar{U}}-(\beta _{10}^{\sigma }+\beta _{11}^{\sigma }(T^{*}-T_{\mathrm{opt}}))}{1+\bar{\bar{U}}} ), \lambda _{2}= -\gamma _{1} \beta ^{(T^{*}- T_{0})}$.

The eigenvalue $\lambda _{1}$ is negative if $g_{0}^{\sigma } (\exp (-b ( \frac{T^{*}-T_{\mathrm{opt}}}{T_{\max } - T_{\mathrm{opt}}} ) )+ \frac{\bar{\bar{U}}-(\beta _{10}^{\sigma }+\beta _{11}^{\sigma }(T^{*}-T_{\mathrm{opt}}))}{1+\bar{\bar{U}}} )< 0$ and positive if $g_{0}^{\sigma } (\exp (-b ( \frac{T^{*}-T_{\mathrm{opt}}}{T_{\max } - T_{\mathrm{opt}}} ) )+ \frac{\bar{\bar{U}}-(\beta _{10}^{\sigma }+\beta _{11}^{\sigma }(T^{*}-T_{\mathrm{opt}}))}{1+\bar{\bar{U}}} )> 0$.

The other eigenvalue $\lambda _{2}$ is negative. Hence the required results are obtained. □

### Lemma 4

*The given equilibrium point* $E^{**}$ *of the fractional*-*order system* (14)*–*(15) *is always locally asymptotically stable*.

### Proof

The Jacobian matrix of system (14)–(15) with respect to $E^{**}$ is

$$\begin{aligned} M_{22}= \begin{bmatrix} b_{11} & b_{12} \\ b_{21} & b_{22}\end{bmatrix}, \end{aligned}$$

where $b_{11}= \frac{- g_{0}^{\sigma }N^{**}}{\gamma _{0}}$, $b_{12}=g_{0}^{\sigma }N^{**} ( \frac{1+\beta _{10}^{\sigma }+\beta _{11}^{\sigma }(T^{*}-T_{\mathrm{opt}})}{(1+U^{**})^{2}} ), b_{21}= -\delta _{2}^{\sigma }U^{**}, b_{22}= -\gamma _{1} \beta ^{(T^{*}- T_{0})}- \delta _{2}^{\sigma }N^{**}$.

The behavior of the eigenvalues is estimated by using Hurwitz’s criteria in the quadratic equation

19 $$\begin{aligned} & \lambda ^{2}+ \lambda \biggl( \frac{- g_{0}^{\sigma }N^{**}}{\gamma _{0}}+ \gamma _{1} \beta ^{(T^{*}- T_{0})}- \delta _{2}^{\sigma }N^{**} \biggr) \\ &\qquad{}+ \biggl(\delta _{2}^{\sigma }U^{**}g_{0}^{\sigma }N^{**} \biggl( \frac{1+\beta _{10}^{\sigma }+\beta _{11}^{\sigma }(T^{*}-T_{\mathrm{opt}})}{(1+U^{**})^{2}} \biggr)+ \frac{g_{0}^{\sigma }N^{**}}{\gamma _{0}}\gamma _{1} \beta ^{(T^{**}- T_{0})}+ \frac{g_{0}^{\sigma }\delta _{2}^{\sigma }N^{{**}^{2}}}{\gamma _{0}} \biggr) \\ &\quad =0. \end{aligned}$$

Using Hurwitz’s criteria, we observe that the eigenvalues $\lambda _{1}, \lambda _{2}$ of the matrix $M_{22}$ are negative if $T^{*} > T_{\mathrm{opt}}$. Thus we get that $E^{**}$ is locally asymptotically stable under the restriction $T^{*}> T_{\mathrm{opt}}$. □

Now for the deformation of fractional-order system (2), we convert it to an equivalent compact form in the case of singular kernels as follows:

20 $$\begin{aligned} \textstyle\begin{cases} {}^{C}D_{t}^{\sigma, \varkappa } N(t) = \mathcal{Q}_{1} (t, N), \\ {}^{C}D_{t}^{\sigma, \varkappa } T(t) = \mathcal{Q}_{2} (t, T), \\ {}^{C}D_{t}^{\sigma, \varkappa } C(t) = \mathcal{Q}_{3} (t, C), \\ {}^{C}D_{t}^{\sigma, \varkappa } Z(t) = \mathcal{Q}_{4} (t, Z), \\ {}^{C}D_{t}^{\sigma, \varkappa } U(t) = \mathcal{Q}_{5} (t, U). \end{cases}\displaystyle \end{aligned}$$

Here $\mathcal{Q}_{1}, \mathcal{Q}_{2}, \mathcal{Q}_{3}, \mathcal{Q}_{4}, \mathcal{Q}_{5}$ are the proposed kernels with respect to the given classes $N, T, C, Z, U$, respectively.

## Fractional-order analysis on the proposed model

### Analysis of the existence and uniqueness of the solution

Proving the existence of the solution for fractional-order systems is always a sensitive part because not all fractional differential equations have their proof of the existence of a solution. In this area a number of works have been done, and lots of researchers work. Here, before deriving the solution of the proposed model, we first prove that the given fractional-order model has a unique solution. We give the results only for the class $N(\zeta )$, and the results are as for the other model classes. So we recall the model equation for *N*,

21a $$\begin{aligned} &{}^{C}D_{\zeta }^{\sigma, \varkappa } N(\zeta ) = \mathcal{Q}_{1}(\zeta,N), \end{aligned}$$

21b $$\begin{aligned} &N(0)= N_{0}, \end{aligned}$$

and the relative Volterra integral equation

22 $$\begin{aligned} N(\zeta )= N(0)+ \frac{{\varkappa }^{1- \sigma }}{\Gamma (\sigma )} \int _{0}^{\zeta }{\xi ^{\varkappa - 1}{\bigl(\zeta ^{\varkappa }- \xi ^{\varkappa }\bigr)}^{\sigma - 1}\mathcal{Q}_{1}( \xi, N)\,d\xi }. \end{aligned}$$

#### Theorem 1

([46] (Existence))

*Let* $0< \sigma \leq 1, N_{0} \in \mathbb{R}, K> 0$, *and* $T^{*} > 0$. *Define* $\mathcal{Q}:= \{(\zeta, N): \zeta \in [0, T^{*}], | N- N_{0} | \leq K\}$, *and let the mapping* $\mathcal{Q}_{1}: \mathcal{Q} \rightarrow \mathbb{R}$ *be continuous*. *Further*, *define* $M:= \sup_{(\zeta, N)\in \mathcal{Q}} |\mathcal{Q}_{1}(\zeta, N)|$ *and*

23 $$\begin{aligned} T= \textstyle\begin{cases} T^{*}& \textit{if } M= 0, \\ \min \{T^{*}, { (\frac{K\Gamma (\sigma +1)\rho ^{\sigma }}{M} )}^{\frac{1}{\sigma }} \}& \textit{otherwise}. \end{cases}\displaystyle \end{aligned}$$

*Then the IVP* (21a)*–*(21b) *has a solution* $N\in C[0, T]$.

#### Lemma 5

([46])

*By considering the result of Theorem *1*a function* $N \in C[0, T]$ *solves the IVP* (21a)*–*(21b) *if and only if it solves the Volterra integral equation* (22).

#### Theorem 2

([46] (Uniqueness))

*Let* $N(0)\in \mathbb{R}, K > 0$, *and* $T^{*} > 0$. *Further*, *let* $0< \sigma \leq 1$ *and* $m= \lceil \sigma \rceil $. *For the set* $\mathcal{Q}$ *given in Theorem *1, *let* $\mathcal{Q}_{1}: \mathcal{Q} \rightarrow \mathbb{R}$ *be a continuous function that satisfies the Lipschitz condition with respect to the second variable*, *that is*,

$$\begin{aligned} \bigl\vert \mathcal{Q}_{1}(\zeta, N_{1}) - \mathcal{Q}_{1}(\zeta, N_{2}) \bigr\vert \leq V \vert N_{1} - N_{2} \vert \end{aligned}$$

*with a constant* $V > 0$ *independent of* $\zeta, N_{1}$, *and* $N_{2}$. *Then the IVP* (21a)*–*(21b) *has a unique solution* $N\in C[0, T]$.

### Numerical solution of the proposed model with application of the generalized predictor–corrector technique

In the last few years, a number of fractional-order numerical schemes have been proposed by the scientists to solve various types of dynamical models. Very recently, the authors of [47] have proposed a new numerical method in the generalized Caputo derivative sense. Here we solve the proposed model with the help of generalized P-C scheme for the solution of the IVP (21a)–(21b) by following the methodology proposed in [44]. Also, we will analyze the stability of the given scheme. In that way, we first recall the above given Volterra integral equation (22), which gives

24 $$\begin{aligned} N(\zeta )= N(0)+ \frac{{\varkappa }^{1- \sigma }}{\Gamma (\sigma )} \int _{0}^{\zeta }{\xi ^{\varkappa - 1}{\bigl(\zeta ^{\varkappa }- \xi ^{\varkappa }\bigr)}^{\sigma - 1}\mathcal{Q}_{1}( \xi, N)\,d\xi }. \end{aligned}$$

Now with supposing that a unique solution exists for the function $\mathcal{Q}_{1}$ on the interval $[0, T]$, we divide the adopted interval $[0, T]$ into *N* unequal subparts $\{[\zeta _{k}, \zeta _{k+1}], k = 0, 1,\ldots, N- 1\}$ using the mesh points

25 $$\begin{aligned} \textstyle\begin{cases} \zeta _{0}= 0, \\ \zeta _{k+1}= {(\zeta ^{\varkappa }_{k}+ h)}^{1/ \varkappa }, \quad k= 0, 1,\ldots, \mathbb{N}- 1, \end{cases}\displaystyle \end{aligned}$$

where $h = \frac{T^{\varkappa }}{\mathbb{N}}$. Now let us try to analyze the approximations $S_{k}, k = 0, 1,\ldots,\mathbb{N}$, to get a numerical solution of the given IVP. Suppose that we have already derived the approximations $N_{j} \approx N(\zeta _{j})\ (j = 1, 2,\ldots, k)$ and want to derive approximations $N_{k+1} \approx N(\zeta _{k+1})$ by means of the integral equation

26 $$\begin{aligned} N(\zeta _{k+1})= N(0)+ \frac{{\varkappa }^{1- \sigma }}{\Gamma (\sigma )} \int _{0}^{\zeta _{k+1}}{ \xi ^{\varkappa - 1}{\bigl(\zeta _{k+1}^{\varkappa }- \xi ^{\varkappa }\bigr)}^{ \sigma - 1} \mathcal{Q}_{1}(\xi, N)\,d\xi }. \end{aligned}$$

By substitution $z = \xi ^{\varkappa }$ we get

27 $$\begin{aligned} N(\zeta _{k+1})= N(0)+ \frac{{\varkappa }^{- \sigma }}{\Gamma (\sigma )} \int _{0}^{\zeta _{k+1}^{\varkappa }}{\bigl(\zeta _{k+1}^{\varkappa }- z\bigr)}^{\sigma - 1}\mathcal{Q}_{1}\bigl(z^{1/ \varkappa }, N \bigl(z^{1/\varkappa }\bigr)\bigr)\,dz, \end{aligned}$$

that is,

28 $$\begin{aligned} N(\zeta _{k+1})= N(0)+ \frac{{\varkappa }^{- \sigma }}{\Gamma (\sigma )}\sum _{j=0}^{k} \int _{\zeta _{j}^{\varkappa }}^{\zeta _{k+1}^{\varkappa }}{\bigl(\zeta _{k+1}^{\varkappa }- z\bigr)}^{\sigma - 1}\mathcal{Q}_{1}\bigl(z^{1/\varkappa }, N \bigl(z^{1/ \varkappa }\bigr)\bigr)\,dz. \end{aligned}$$

Now, to simulate the right-side of Eq. (28), applying the trapezoidal quadrature rule with respect to the weight function ${(\zeta _{k+1}^{\varkappa }- z)}^{\sigma - 1}$ and shifting the function $G_{1}(z^{1/\varkappa }, N(z^{1/\varkappa }))$ by its piecewise linear interpolant with nodes $\zeta ^{\varkappa }_{j} \ (j = 0, 1,\ldots,k + 1)$, we get

29 $$\begin{aligned} \begin{aligned} & \int _{\zeta _{j}^{\varkappa }}^{\zeta _{k+1}^{\varkappa }}{\bigl( \zeta _{k+1}^{\varkappa }- z\bigr)}^{\sigma - 1}\mathcal{Q}_{1}\bigl(z^{1/ \varkappa }, N \bigl(z^{1/\varkappa }\bigr)\bigr)\,dz \\ &\quad\approx \frac{h^{\sigma }}{\sigma (\sigma + 1)} \bigl[ \bigl({(k- j)}^{\sigma + 1}- {(k- j- \sigma )} {(k- j+ 1)}^{\sigma } \bigr) \\ & \qquad{}\times G_{1}\bigl(\zeta _{j}, N(\zeta _{j}) \bigr) + \bigl({(k- j+ 1)}^{ \sigma +1}- (k- j+ \sigma + 1){(k- j)^{\sigma }} \bigr)\mathcal{Q}_{1}\bigl( \zeta _{j+1}, N(\zeta _{j+1})\bigr) \bigr]. \end{aligned} \end{aligned}$$

So, fitting the above-proposed approximations in Eq. (28), we establish the corrector term for $N(\zeta _{k+1}), k = 0, 1,\ldots,\mathbb{N} - 1$:

30 $$\begin{aligned} N(\zeta _{k+1})\approx N(0)+ \frac{{\varkappa }^{- \sigma }h^{\sigma }}{\Gamma (\sigma + 2)}\sum _{j=0}^{k}a_{j, k+1} \mathcal{Q}_{1}\bigl(\zeta _{j}, N(\zeta _{j}) \bigr)+ \frac{\varkappa ^{-\sigma }h^{\sigma }}{\Gamma (\sigma +2)}\mathcal{Q}_{1}\bigl( \zeta _{k+1}, N(\zeta _{k+1})\bigr), \end{aligned}$$

where

31 $$\begin{aligned} a_{j, k+1}= \textstyle\begin{cases} k^{\sigma +1}- (k- \sigma ){(k+ 1)}^{\sigma } &\text{if } j=0, \\ {(k- j+ 2)}^{\sigma + 1}+ {(k- j)}^{\sigma +1}- 2{(k- j+1)}^{\sigma +1} &\text{if } 1\leq j\leq k. \end{cases}\displaystyle \end{aligned}$$

The final task for our solution is changing the quantity $N(\zeta _{k+1})$ on the right-hand side of formula (30) with the predictor value $N^{P}(\zeta _{k+1})$, which can be calculated by applying the one-step Adams–Bashforth technique to the integral equation (27). In this case, by changing the mapping $\mathcal{Q}_{1}(z^{1/\varkappa }, N(z^{1/\varkappa }))$ by the quantity $\mathcal{Q}_{1}(\zeta _{j}, N(\zeta _{j}))$ at each integral in Eq. (28) we get

32 $$\begin{aligned} \begin{aligned} N^{P}(\zeta _{k+1})& \approx N(0)+ \frac{\varkappa ^{- \sigma }}{\Gamma (\sigma )}\sum _{j=0}^{k} \int _{\zeta _{j}^{\varkappa }}^{\zeta _{j+1}^{\varkappa }}{\bigl(\zeta _{k+1}^{\varkappa }- z\bigr)}^{\sigma - 1}\mathcal{Q}_{1}\bigl(\zeta _{j}, N( \zeta _{j})\bigr)\,dz \\ & = N(0)+ \frac{\rho ^{- \sigma }h^{\sigma }}{\Gamma (\sigma +1)}\sum_{j=0}^{k} \bigl[{(k+1- j)}^{\sigma }-{(k- j)}^{\sigma }\bigr]\mathcal{Q}_{1} \bigl( \zeta _{j}, N(\zeta _{j})\bigr). \end{aligned} \end{aligned}$$

So our P-C method for deriving the approximations $N_{k+1} \approx N(\zeta _{k+1})$ is totally evaluated by the formula

33 $$\begin{aligned} N_{k+1}\approx N(0)+ \frac{{\varkappa }^{- \sigma }h^{\sigma }}{\Gamma (\sigma + 2)}\sum _{j=0}^{k}a_{j, k+1}\mathcal{Q}_{1}( \zeta _{j}, N_{j})+ \frac{\varkappa ^{-\sigma }h^{\sigma }}{\Gamma (\sigma +2)}\mathcal{Q}_{1} \bigl( \zeta _{k+1}, N^{P}_{k+1}\bigr), \end{aligned}$$

where $N_{j} \approx N(\zeta _{j}), j = 0, 1,\ldots,k$, and the predicted value $N^{P}_{k+1}\approx N^{P}(\zeta _{k+1})$ can be simulated as mentioned in Eq. (32) with the terms $a_{j,k+1}$ estimated according to (31).

Therefore the derivation for the approximate solution of the proposed system (2) is derived successfully and defined by the following equations:

34 $$\begin{aligned} \begin{aligned} & N_{k+1}\approx N(0)+ \frac{{\varkappa }^{- \sigma }h^{\sigma }}{\Gamma (\sigma + 2)}\sum_{j=0}^{k}a_{j, k+1} \mathcal{Q}_{1}(\zeta _{j}, N_{j})+ \frac{\varkappa ^{-\sigma }h^{\sigma }}{\Gamma (\sigma +2)}\mathcal{Q}_{1}\bigl( \zeta _{k+1}, N^{P}_{k+1}\bigr), \\ & T_{k+1}\approx T(0)+ \frac{{\varkappa }^{- \sigma }h^{\sigma }}{\Gamma (\sigma + 2)}\sum _{j=0}^{k}a_{j, k+1}\mathcal{Q}_{2}( \zeta _{j}, T_{j})+ \frac{\varkappa ^{-\sigma }h^{\sigma }}{\Gamma (\sigma +2)}\mathcal{Q}_{2} \bigl( \zeta _{k+1}, T^{P}_{k+1}\bigr), \\ & C_{k+1}\approx C(0)+ \frac{{\varkappa }^{- \sigma }h^{\sigma }}{\Gamma (\sigma + 2)}\sum _{j=0}^{k}a_{j, k+1}\mathcal{Q}_{3}( \zeta _{j}, C_{j})+ \frac{\varkappa ^{-\sigma }h^{\sigma }}{\Gamma (\sigma +2)}\mathcal{Q}_{3} \bigl( \zeta _{k+1}, C^{P}_{k+1}\bigr), \\ & Z_{k+1}\approx Z(0)+ \frac{{\varkappa }^{- \sigma }h^{\sigma }}{\Gamma (\sigma + 2)}\sum _{j=0}^{k}a_{j, k+1}\mathcal{Q}_{4}( \zeta _{j}, Z_{j})+ \frac{\varkappa ^{-\sigma }h^{\sigma }}{\Gamma (\sigma +2)}\mathcal{Q}_{4} \bigl( \zeta _{k+1}, Z^{P}_{k+1}\bigr), \\ & U_{k+1}\approx U(0)+ \frac{{\varkappa }^{- \sigma }h^{\sigma }}{\Gamma (\sigma + 2)}\sum _{j=0}^{k}a_{j, k+1}\mathcal{Q}_{5}( \zeta _{j}, U_{j})+ \frac{\varkappa ^{-\sigma }h^{\sigma }}{\Gamma (\sigma +2)}\mathcal{Q}_{5} \bigl( \zeta _{k+1}, U^{P}_{k+1}\bigr), \end{aligned} \end{aligned}$$

where

35 $$\begin{aligned} & N^{P}(\zeta _{k+1}) \approx N(0)+ \frac{\varkappa ^{- \sigma }h^{\sigma }}{\Gamma (\sigma +1)}\sum _{j=0}^{k}\bigl[{(k+1- j)}^{\sigma }-{(k- j)}^{\sigma }\bigr]\mathcal{Q}_{1}\bigl( \zeta _{j}, N( \zeta _{j})\bigr), \\ & T^{P}(\zeta _{k+1}) \approx T(0)+ \frac{\varkappa ^{- \sigma }h^{\sigma }}{\Gamma (\sigma +1)}\sum _{j=0}^{k}\bigl[{(k+1- j)}^{\sigma }-{(k- j)}^{\sigma }\bigr]\mathcal{Q}_{2}\bigl( \zeta _{j}, T(\zeta _{j})\bigr), \\ & C^{P}(\zeta _{k+1}) \approx C(0)+ \frac{\varkappa ^{- \sigma }h^{\sigma }}{\Gamma (\sigma +1)}\sum _{j=0}^{k}\bigl[{(k+1- j)}^{\sigma }-{(k- j)}^{\sigma }\bigr]\mathcal{Q}_{3}\bigl( \zeta _{j}, C(\zeta _{j})\bigr), \\ & Z^{P}(\zeta _{k+1}) \approx Z(0)+ \frac{\varkappa ^{- \sigma }h^{\sigma }}{\Gamma (\sigma +1)}\sum _{j=0}^{k}\bigl[{(k+1- j)}^{\sigma }-{(k- j)}^{\sigma }\bigr]\mathcal{Q}_{4}\bigl( \zeta _{j}, Z(\zeta _{j})\bigr), \\ & U^{P}(\zeta _{k+1}) \approx U(0)+ \frac{\varkappa ^{- \sigma }h^{\sigma }}{\Gamma (\sigma +1)}\sum _{j=0}^{k}\bigl[{(k+1- j)}^{\sigma }-{(k- j)}^{\sigma }\bigr]\mathcal{Q}_{5}\bigl( \zeta _{j}, U(\zeta _{j})\bigr). \end{aligned}$$

#### Method stability

##### Theorem 3

*Let* $\mathcal{Q}_{1}(\zeta, N), \mathcal{Q}_{2}(\zeta, T), \mathcal{Q}_{3}( \zeta, C), \mathcal{Q}_{4}(\zeta, Z), \mathcal{Q}_{5}(\zeta, U)$ *satisfy the Lipschitz condition*, *and let* $N_{j}, T_{j}, C_{j}, Z_{j}, U_{j} \ (j = 1,\ldots, k + 1)$ *be approximate solutions of the derived P*-*C method* (34) *and* (35), *respectively*. *Then the proposed numerical algorithm* (34)*–*(35) *is conditionally stable*.

##### Proof

Let $\tilde{N_{0}}, \tilde{N_{j}} (j= 0,\ldots, k+1)$, and $\tilde{N_{k+1}^{P}} \ (k= 0,\ldots,\mathcal{N}-1)$ be perturbations of $N_{0}, N_{j}$, and $N_{k+1}^{P}$, respectively. Then the given below perturbation equations are estimated with the help of Eqs. (34) and (35).

36 $$\begin{aligned} \tilde{N^{P}_{k+1}}= \tilde{N_{0}}+ \frac{\varkappa ^{- \sigma }h^{\sigma }}{\Gamma (\sigma +1)}\sum_{j=0}^{k}b_{j, k+1} \bigl(\mathcal{Q}_{1}(\zeta _{j}, N_{j}+ \tilde{N_{j}})- \mathcal{Q}_{1}(\zeta _{j},N_{j}) \bigr), \end{aligned}$$

where $b_{j, k+1}= [{(k+1- j)}^{\sigma }-{(k- j)}^{\sigma }]$,

37 $$\begin{aligned} \begin{aligned} \tilde{N_{k+1}}={}& \tilde{N_{0}}+ \frac{\varkappa ^{- \sigma }h^{\sigma }}{\Gamma (\sigma +2)}\bigl(\mathcal{Q}_{1}\bigl( \zeta _{k+1}, N^{P}_{k+1}+\tilde{N^{P}_{k+1}}\bigr)- \mathcal{Q}_{1}\bigl( \zeta _{k+1}, N^{P}_{k+1} \bigr)\bigr)+ \frac{\varkappa ^{- \sigma }h^{\sigma }}{\Gamma (\sigma +2)} \\ & {}\times \sum_{j=0}^{k} a_{j, k+1} \bigl(\mathcal{Q}_{1}(\zeta _{j}, N_{j}+ \tilde{N_{j}})- \mathcal{Q}_{1}(\zeta _{j}, N_{j})\bigr). \end{aligned} \end{aligned}$$

Using the Lipschitz condition, we obtain

38 $$\begin{aligned} \vert \tilde{N_{k+1}} \vert \leq \zeta _{0}+ \frac{\varkappa ^{- \sigma }h^{\sigma }m_{1}}{\Gamma (\sigma +2)} \Biggl( \bigl\vert \tilde{N^{P}_{k+1}} \bigr\vert +\sum_{j=1}^{k} a_{j, k+1} \vert \tilde{N_{j}} \vert \Biggr), \end{aligned}$$

where $\zeta _{0}= \max_{0\leq k\leq \mathcal{N}}\{|\tilde{N_{0}}|+ \frac{\varkappa ^{-\sigma }h^{\sigma }m_{1} a_{k,0}}{\Gamma (\sigma + 2)}| \tilde{N_{0}}|\}$. Also, from Eq. (3.18) in [45] we derive

39 $$\begin{aligned} \bigl\vert \tilde{N^{P}_{k+1}} \bigr\vert \leq \eta _{0}+ \frac{\varkappa ^{- \sigma }h^{\sigma }m_{1}}{\Gamma (\sigma +1)}\sum_{j=1}^{k}b_{j, k+1} \vert \tilde{N_{j}} \vert , \end{aligned}$$

where $\eta _{0}= \max_{0\leq k\leq N}\{|\tilde{N_{0}}|+ \frac{\varkappa ^{- \sigma }h^{\sigma }m_{1} b_{k,0}}{\Gamma (\sigma +1)}| \tilde{N_{0}}|\}$. Substituting $|\tilde{N^{P}_{k+1}}|$ from Eq. (39) into Eq. (38) results in

40 $$\begin{aligned} \vert \tilde{N_{k+1}} \vert \leq{}& \sigma _{0}+ \frac{\varkappa ^{- \sigma }h^{\sigma }m_{1}}{\Gamma (\sigma +2)} \Biggl( \frac{\varkappa ^{- \sigma }h^{\sigma }m_{1}}{\Gamma (\sigma +1)}\sum _{j=1}^{k}b_{j, k+1} \vert \tilde{N_{j}} \vert +\sum_{j=1}^{k} a_{j, k+1} \vert \tilde{N_{j}} \vert \Biggr), \end{aligned}$$

41 $$\begin{aligned} \leq{}& \sigma _{0}+ \frac{\varkappa ^{- \sigma }h^{\sigma }m_{1}}{\Gamma (\sigma +2)}\sum _{j=1}^{k} \biggl( \frac{\varkappa ^{- \sigma }h^{\sigma }m_{1}}{\Gamma (\sigma +1)}b_{j, k+1}+ a_{j, k+1} \biggr) \vert \tilde{N_{j}} \vert , \end{aligned}$$

42 $$\begin{aligned} \leq{}& \sigma _{0}+ \frac{\varkappa ^{-\sigma }h^{\sigma }m_{1} C_{\sigma, 2}}{\Gamma (\sigma + 2)} \sum _{j=1}^{k}(k+ 1- j)^{\sigma - 1} \vert \tilde{N_{j}} \vert , \end{aligned}$$

where $\sigma _{0}= \max \{\zeta _{0}+ \frac{\varkappa ^{-\sigma }h^{\sigma }m_{1} a_{k+1,k+1}}{\Gamma (\sigma + 2)} \eta _{0}\}$, $C_{\sigma,2}$ is a positive constant depending on *σ* (by Lemma 1), and *h* is assumed to be small enough. Using Lemma 2, we have $|\tilde{N_{k+1}}|\leq C \sigma _{0}$. which concludes the proof. □

## Experimental simulations

After finishing all necessary theoretical analysis, we start to perform some experimental calculations to show the correctness of our results. We use *Mathematica* software for performing the number of graphs. For the case of interior equilibrium point $E^{*}(U^{*}, Z^{*}, C^{*}, T^{*}, N^{*})$, we use the following set of parameter values: $g_{0}= 0.9, \beta _{10}= 0.03, \beta _{11}= 0.001, T_{\mathrm{opt}}= 24, \gamma _{0}= 150, D_{s_{0}}= 4, A_{0}= 0.2, w= 2.10, \zeta _{1}= 3.5, O_{c}= 1.10, \Lambda _{1}= 0.66, \Lambda = 0.4, \gamma _{1}= 2, \delta _{2}= 0.2, \zeta = 0.0019, \beta = 1.024, \gamma =4, z_{0}= 10.10, T_{10}= 14.50, \delta _{1}= 0.1, T_{0}= 20, b= 1.30, T_{\max }= 35, N(0)= 10, T(0)= 28, C(0)= 1, Z(0)= 1.2, U(0)= 0.25$. Here we observe that for the case of fractional order $\sigma =1$ (when the model behaves like an integer-order system), the authors of [1] have calculated the value of the interior equilibrium point $E^{*}(0.1023, 0.7534, 2.0000, 26.3818, 122.7088)$ and then, in this case, have specified the constraints for the solution boundedness, equilibrium point $E^{*}$ stability, and positivity of the solution. Our target is to explore the dynamics of all model classes with respect to the interior equilibrium points at different fractional-order values *σ*.

**Figure 2:**
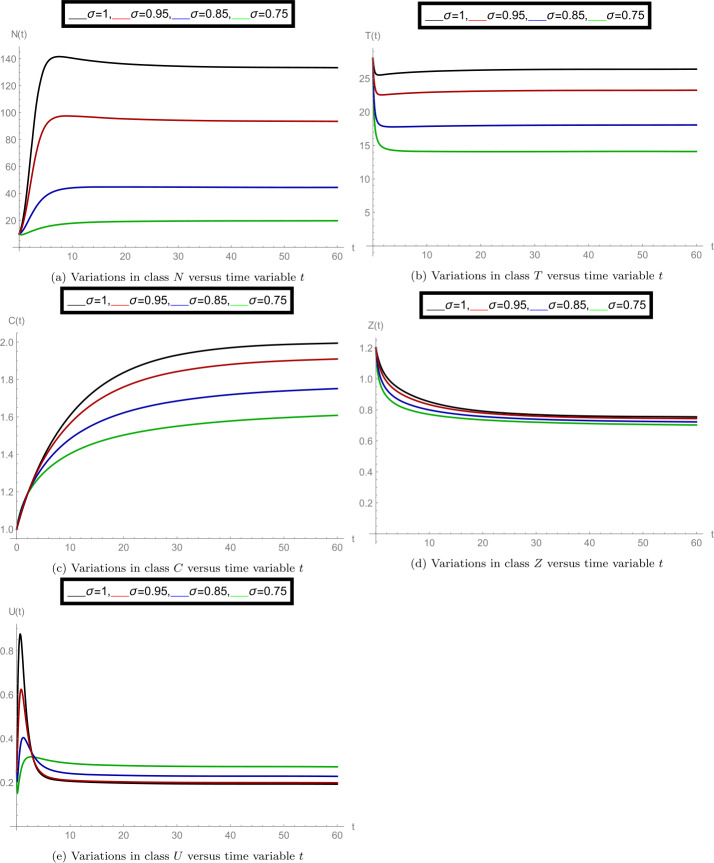
Separate plots of all model classes at various fractional-order values *σ* for the case of interior equilibrium point $E^{*}(U^{*}, Z^{*}, C^{*}, T^{*}, N^{*})$

**Figure 3:**
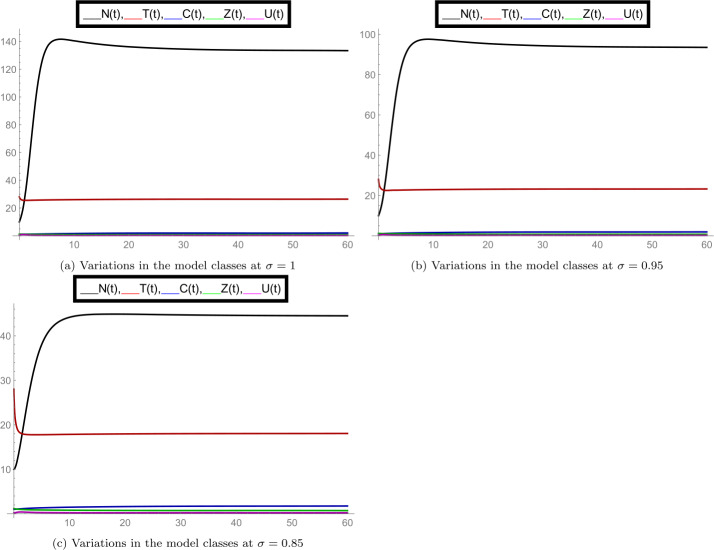
Mixed plots of all model classes at fractional-order values $\sigma = 1, 0.95, 0.85$ for the case of interior equilibrium point $E^{*}(U^{*}, Z^{*}, C^{*}, T^{*}, N^{*})$

In the set of Fig. 2, we observed the nature of all model classes separately at different fractional-order values *σ*. In subfigure 2(a) the dynamics of density of aquatic population *N* is plotted at $\sigma = 1, 0.95, 0.85, 0.75$. Here we observed that at $\sigma =1$ the numerically calculated equilibrium point is satisfied for class *N* and also at other values of order *σ*, it changed simultaneously. Similarly, subfigure 2(b) shows the average water temperature of the species (class *T*), subfigure 2(c) shows the concentration of greenhouse gases (class *C*), subfigure 2(d) shows the ozone concentration (class *Z*), and subfigure 2(e) shows the dynamics of dissolved oxygen concentration (class *U*). The simultaneous changes in the given model classes at particular values of *σ* can be seen from the set of Fig. 3. Overall, we observed that when the fractional-order *σ* changes, the dynamics of the model, along with interior equilibrium point changes, justifies the importance of the fractional-order model.

As investigated above, now we consider the case of boundary equilibrium point $\bar{E}= (\bar{U}, \bar{Z}, \bar{C}, \bar{T}, \bar{N})$. In this case, we consider the following parameter values: $g_{0}= 0.9, \beta _{10}= 0.03, \beta _{11}= 0.50, T_{\mathrm{opt}}= 24, \gamma _{0}= 150, D_{s_{0}}= 4, A_{0}= 0.7, w= 2.1710, \zeta _{1}= 3.5, O_{c}= 1.10, \Lambda _{1}= 0.66, \Lambda = 0.4, \gamma _{1}= 1, \delta _{2}= 0.6, \zeta = 0.11, \beta = 1.024, \gamma =4, z_{0}= 10.10, T_{10}= 14.50, \delta _{1}= 0.1, T_{0}= 20, b= 1.30, T_{\max }= 35, N(0)= 10, T(0)= 28, C(0)= 1, Z(0)= 1.2, U(0)= 0.25$. For the given parameter weights, the value of boundary equilibrium point $\bar{E}= (\bar{U}, \bar{Z}, \bar{C}, \bar{T}, \bar{N})$ at fractional-order $\sigma =1$ (when the model behaves like an integer-order system given in [1]) is $\bar{E}(0.0474, 0.3179, 7.0, 30.0216, 0)$. In that integer-order case, the boundary equilibrium point is linearly asymptotically stable.

**Figure 4:**
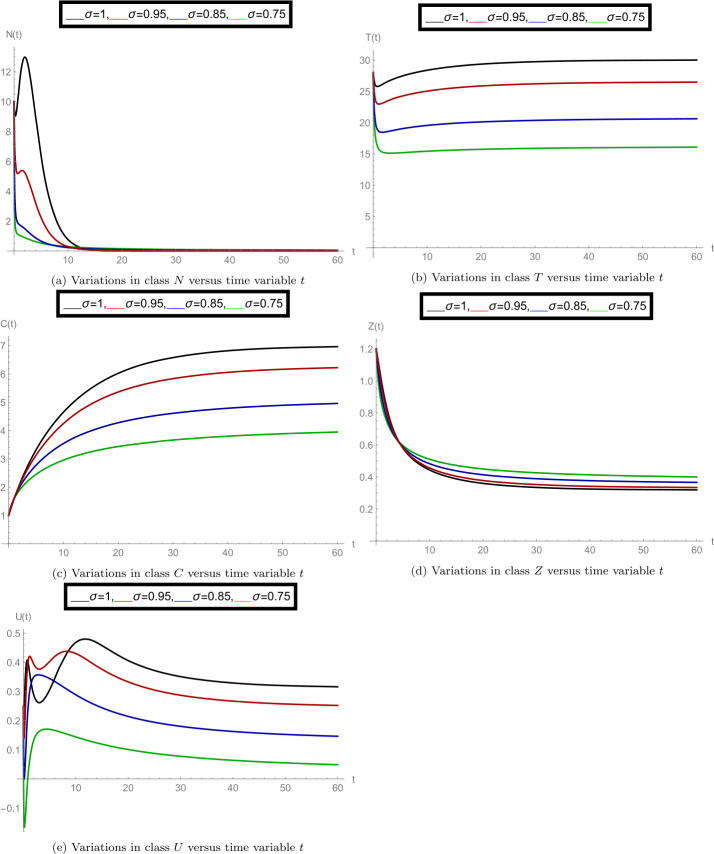
Separate plots of all model classes at various fractional-order values *σ* for the case of boundary equilibrium point $\bar{E}= (\bar{U}, \bar{Z}, \bar{C}, \bar{T}, \bar{N})$

For the noninteger-order observations, in the set of Fig. 4, we analyzed the nature of proposed model classes separately at various fractional-order values *σ*. In subfigure 4(a), the dynamics of density of aquatic population *N* is plotted at $\sigma = 1, 0.95, 0.85, 0.75$. Here we can see that for $\sigma =1$, the numerically calculated equilibrium point is satisfied for population *N* and that at other values of order *σ*, it changes simultaneously. Following the same way, subfigure 4(b) specifies the average water temperature of the species (class *T*), subfigure 4(c) demonstrates the concentration of greenhouse gases (class *C*), subfigure 4(d) shows the ozone concentration (class *Z*), and subfigure 4(e) shows the dynamics of dissolved oxygen concentration (class *U*).

**Figure 5:**
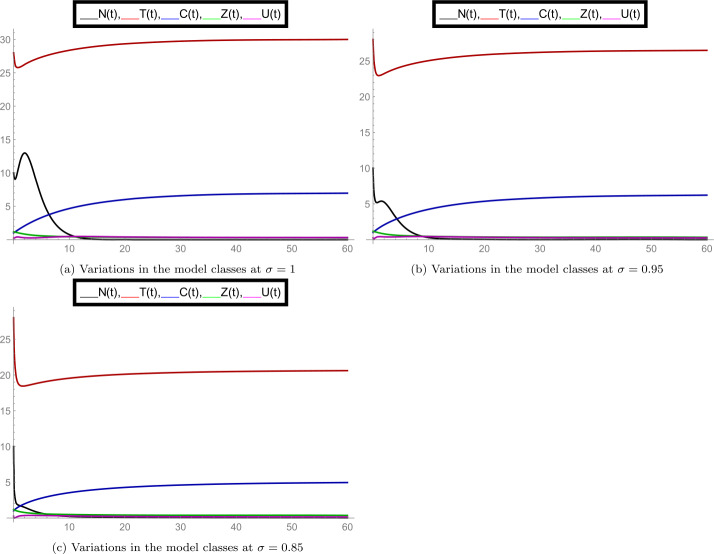
Mixed plots of all model classes at fractional-order values $\sigma = 1, 0.95, 0.85$ for the case of boundary equilibrium point $\bar{E}= (\bar{U}, \bar{Z}, \bar{C}, \bar{T}, \bar{N})$

The simultaneous changes in the given model classes at particular value of *σ* can be analyzed from the set of Fig. 5. Overall, we can see that when the fractional-order *σ* changes, the dynamics of the model changes along with boundary equilibrium point, which satisfies the role of fractional-order operator.

From the above given experimental analysis we see that the fractional-order dynamics with memory effects is much stronger than the integer-order dynamics. Here we have more varieties to understand the structure of the proposed ecosystem dynamics at various fractional-order values along with different values of equilibrium points. The modified Caputo fractional derivative is fully suitable to simulate the novel results with the help of given fractional-order model.

## Conclusion

In our study, we have simulated a novel fractional-order mathematical system to study the prelude of deteriorating quality of water because of greenhouse gases on the population of aquatic animals. It has been shown in the given system that greenhouse gases raise the temperature of water, and because of this reason, the dissolved oxygen level goes down, and also the rate of circulation of disintegrated oxygen by the species rises, which causes a decrement in the density of aquatic species. We have used a new generalized Caputo-type fractional-order derivative to simulate the given dynamics. Equilibrium points for the given fractional model have been calculated, and important discussion on the asymptotic stability of the equilibria of a new autonomous system has been evaluated. We have reminded some important results to prove the existence of unique solution for the fractional-order cases. For finding the numerical solution of the given system, we used a generalized predictor–corrector algorithm in the sense of the new generalized Caputo derivative and also justified the stability of the technique. To prove the importance and correctness of the numerically simulated results, we have performed a number of graphs at different fractional-order values. The given derivative and algorithm work very well to understand the dynamics of the given model. From this study the effects of greenhouse gases and hypoxia on the population of aquatic species can be clearly understood with memory effects. For the future scope, the given ecosystem can be further solved by any other fractional-order derivatives. Also, some new mathematical models can be proposed to simulate the structure of given real-world problems.

## Data Availability

The data used in this study are mentioned/available in the manuscript.
